# 

**Published:** 1934

**Authors:** 


### The Medical Library of the University of Bristol,

WITH WHICH IS INCORPORATED

**The Library of the Bristol Medico-Chirurgical Society.**

---

*The following donations have been received since the publication of the list in June,* 1934.

September, 1934.

| | |
|---|---|
| American Laryngological, Rhinological and Otological Society, Inc. (1) .. .. .. | 2 volumes. |
| J. Michell Clarke Fund (2) .. .. .. | 1 volume. |
| Geneva University (3) .. .. .. .. | 1 ,, |
| Professor E. W. Hey Groves (4) .. .. | 1 ,, |
| Rt. Hon. Henry Hobhouse (5) .. .. .. | 1 ,, |
| Dr. J. E. R. McDonagh (6) .. .. .. | 1 ,, |
| Professor A. Rendle Short (7) .. .. .. | 1 ,, |
| The late Dr. W. Alexander Smith (8) .. .. | 46 volumes. |
| Dr. M. F. Staniland (9) .. .. .. .. | 1 volume. |

Unbound periodicals have also been received from Professor E. W. Hey Groves and Dr. C. Bruce Perry.

---

### THE ONE HUNDRED AND FORTY-EIGHTH LIST OF BOOKS.

The figures in round brackets refer to the figures after the names of the donors. The books to which no such figures are attached have either been bought from the Library Fund or received through the *Journal.*

| | | | | |
|---|---|---|---|---|
| **Alexis** .. .. .. .. .. | *The Secrets of Alexis* .. .. .. .. .. | | (9) | 1615 |
| **Arbuthnot, J.** .. .. | *The Nature of Aliments* .. | 2nd Ed. | (8) | 1732 |
| **Barlow, J. W.** .. .. | *Doctors at War* .. .. .. .. .. .. | | (8) | 1914 |
| **Bennet, J. H.** .. .. | *Winter and Spring on the Shores of the Mediterranean* .. .. .. | 5th Ed. | (8) | 1875 |
| **Brodie, B. E.** .. .. | *Autobiography of the late Sir B. C. Brodie* | | (8) | 1865 |
| **Broster, L. R., and Vines, H. W. C.** | *The Adrenal Cortex* .. .. | | | 1933 |
| **Buchan, W.** .. .. .. | *Domestic Medicine* .. .. .. .. | 9th Ed. | (8) | 1785 |
| **Calthrop, L. C. E.** .. | *Hydrotherapy and Physiotherapy* .. .. | | | 1931 |
| **Cappadocis Aretaei** .. | *Medici Insignis ac Vetustissimi* | Lib. 7 | (8) | 1552 |

| | | | | |
|---|---|---|---|---|
| Central Control Board | *Alcohol : Its Action on Human Organism* | | (8) | 1918 |
| Claridge, R. T. | *Hydrotherapy* | 3rd Ed. | (8) | 1842 |
| Clendening, L. | *Behind the Doctor* | | | 1933 |
| Cormack, J. R. | *Clinical Studies.* Vols. I. and II. | | (8) | 1876 |
| Curtis, J. G. | *Harvey's Views on the Use and Circulation of Blood* | | (8) | 1915 |
| Despard, L. L. | *Text-book of Massage and Remedial Gymnastics* | 3rd Ed. | | 1932 |
| Drewitt, F. D. | *Life of Edward Jenner* | 2nd Ed. | | 1933 |
| Evans, G. | *Essays on Chronic and Familial Syphilis* | | | 1934 |
| Fitzsimmons, F. W. | *The House-fly* | | (8) | 1915 |
| Fresenius, C. R. | *Quantitative Chemical Analysis* | 6th Ed. | (8) | 1873 |
| Frey, Sigurd | *Die Embolie* | | (4) | 1933 |
| Gaskell, W. H. | *The Involuntary Nervous System* | | (8) | 1916 |
| Gehuchten, P. van | *La Pathologie du Système Pallido-strié* | | | 1930 |
| Gillett, H. T. | *Vaccine Therapy* | | | 1933 |
| Guppy, J. | *A Litil Boke for the Pestilence* | | (8) | 1910 |
| Hammond, W. A. | *Lectures on Venereal Diseases* | | (8) | 1864 |
| Hartmann, Franz | *Paracelsus* | 2nd Ed. | (8) | [n.d.] |
| Hilton, J. | *Mechanical and Physiological Rest* | | (8) | 1863 |
| Hobhouse, E., ed. | *Diary of a West Country Physician* | | (5) | 1934 |
| Horsley, V., and Sturge, M.D. | *Alcohol and the Human Body* | | (8) | 1907 |
| Humphry, G. H. | *The Hunterian Oration* | | (8) | 1879 |
| Jenner, E. | *Further Observations on the Variolæ Vaccinæ* | | (8) | 1799 |
| Keen, W. W. | *Animal Experimentation and Medical Progress* | | (8) | 1914 |
| [Kitchiner, W.] | *The Art of Life* | 3rd Ed. | (8) | 1822 |
| Lawrence, R. M. | *Primitive Psycho-Therapy and Quackery* | | (8) | 1910 |
| Lewes, C. L. | *Dr. Southwood Smith* | | (8) | 1898 |
| Liston, R. | *Practical Surgery* | 3rd Ed. | (8) | 1840 |
| McDonagh, J. E. R. | *Nature of Disease Journal.* Vol. III. | | (6) | 1934 |
| Macdonald, J. D. | *Microscopical Examination of Drinking Water* | | (8) | 1875 |
| Mackie, T. J., and McCartney, J. E. | *Introduction to Practical Bacteriology* | 4th Ed. | | 1934 |
| Macnamara, N. C. | *Instinct and Intelligence* | | (8) | 1915 |
| Maudsley, H. | *Natural Causes and Supernatural Seemings* | 2nd Ed. | (8) | 1887 |
| Mayou, M. S. | *Diseases of the Eye* | 4th Ed. | | 1933 |
| Mead, R. | *Discourse on the Plague* | 9th Ed. | (8) | 1744 |
| Mead, R. | *De imperio solis ac limæ in corpora humana et morbis inda oriundis* | | (8) | 1704 |
| Monrad-Krohn, G. H. | *Clinical Examination of the Nervous System* | 6th Ed. | | 1933 |
| *Nomenclature of Diseases* | | 6th Ed. | (8) | 1931 |
| Oliver, Sir T. | *Lead Poisoning* | | (8) | 1914 |
| O'Sullivan, F. | *Miners' Nystagmus* | | | 1933 |
| Paget, G. E. | *Harveian Oration* | | (8) | 1866 |
| Plowman, C. F., and Dearden, W. F. | *Fighting the Fly Peril* | | (8) | 1915 |

| | | | | |
|---|---|---|---|---|
| **Pomme, P.** | *Traité des affections vaporeuses* | 3rd Ed. | (8) | 1767 |
| **Pott, P.** | *Chirurgical Works* | | (8) | 1775 |
| **Pringle, J.** | *Diseases of the Army* | 3rd Ed. | (8) | 1761 |
| *Queen Charlotte's Textbook of Obstetrics* | | 3rd Ed. | (2) | 1933 |
| **Ranke, J.** | *Physiologie des Menschen* | 3rd Ed. | (8) | 1875 |
| **Raynaud, M.** | *Les Médecins au temps de Molière* | 2nd Ed. | (8) | 1863 |
| **Ridley, F., and Sorsby, A.** | *Guide to Fundus Appearances* | | | 1933 |
| **Roullet, M.** | *La détermination du cercle des personnes assurées—thèse* | | (3) | 1928 |
| **Roussy, G., and Lhermitte, J.** | *Psychoneuroses of War* | | (8) | 1918 |
| **Schenck, F., and Gurber, A.** | *Leitfaden der Physiologie des Menschen* | | (8) | 1908 |
| **Searle, C.** | *Life, Health and Disease* | | (8) | 1846 |
| **Smellie, W.** | *Obstetric Plates* | | (8) | 1848 |
| **Smith, G. S. Graham-** | *Flies in Relation to Disease* | | (8) | 1913 |
| **Thomas, E. W. C.** | *Synopsis of Public Health* | | | 1932 |
| **Vazifdar, N. J.** | *Physiology of the Central Nervous System and Special Senses* | 6th Ed. | (7) | 1934 |
| **Williamson, B.** | *Vital Cardiology* | | | 1934 |
| **Wise, F., and others, eds.** | *Dermatology, Syphilis and Urology* | | | 1931 |
| **Wright, S.** | *Applied Physiology* | 5th Ed. | | 1934 |

---

TRANSACTIONS, REPORTS, JOURNALS, ETC.

| | | |
|---|---|---|
| American Laryngological, Rhinological and Otological Society, Inc. Transactions of the 38th and 39th Annual Meetings, 1932 and 1933 | (1) | 1932–3 |
| British Medical Directory | (8) | 1853 |
| Collected Papers of the Mayo Clinic. Vol. 25 (1933) | | 1934 |
| Medical Society's Transactions. Vol. LVII. | | 1934 |
| Surgical Clinics of North America. Vol. 14. No. 3 | | 1934 |

---

### Publications Received

From FÉLIX ALCAN :
*L'Opération de Bassini.* Par A. CATTERINA.

From EDWARD ARNOLD & CO. :
*Diseases of Women.* 5th Ed. By TEN TEACHERS. Price 18s.

From JOHN BALE, SONS & DANIELSSON :
*Birth Control To-day.* By MARIE C. STOPES, D.SC. Price 5s.

From CASSELL & CO. LTD. :
*Diseases of the Skin.* By S. E. DORE AND J. L. FRANKLIN. Price 10s. 6d.

From WILLIAM HEINEMANN LTD. :

*Stand Up and Slim Down.* 6th Ed. By ETTIE A. HORNIBROOK. Price 6s.

From H. K. LEWIS & CO. LTD.:

*Common Skin Diseases.* 2nd Ed. By A. C. ROXBURGH, M.A., M.D., B.CH. Price 16s.

*Pocket Pronouncing Medical Dictionary.* 10th Ed. By G. M. GOULD, A.M., M.D. Price 10s. 6d.

*Constitution and Its Reaction in Health.* By T. E. HAMMOND. Price 7s. 6d.

*Modern Advances in Diseases of the Throat.* By A. MILLER. Price 10s. 6d.

*Students' Handbook of Clinical Electrocardiography.* By WILLIAM EVANS, M.D. Price 5s.

*Murrell's What to do in Cases of Poisoning.* By P. HANNILL, M.D., D.Sc. 14th Ed. Price 21s.

From OXFORD UNIVERSITY PRESS:

*Physics for Medical Students.* By J. S. ROGERS, B.A., M.Sc. Price 11s. 6d.

*Industrial Maladies.* By T. LEGGE, M.D. Price 12s. 6d.

From JOHN WRIGHT & SONS LTD.:

*Massage and Remedial Exercises.* By NOËL M. TIDY. Price 15s.

*Diffuse Sclerosis.* By L. BOUMAN, M.D. Price 15s.

*A Synopsis of Medicine.* By H. LETHEBY TIDY, M.A., M.D., B.CH. 6th Ed. Price 21s.

### Local Medical Notes

**University of Bristol.**—Honorary Degrees: LL.D.—Dr. George Parker; D.Sc.—Sir Robert Muir; M.D.—Dr. Patrick Watson-Williams.

EXAMINATION RESULTS.

Students of the University have recently passed the following examinations :—

M.B., CH.B.—*First Examination:* Catherine M. Addy, D. L. Bayley, Mary M. Brown, J. N. P. Davies, J. L. Elliott, Joan M. Foss, Emily G. Hamlyn, F. R. Hurford, G. W. E. Manners, N. E. Melling, Joyce S. Miller, P. S. Robinson, Jeanette D. Shed, S. J. Silvey, P. R. H. Slade, J. E. Smith, A. A. Steinberg, Edith M. Wagstaff, T. H. White.

B.D.S.—*First Examination:* D. R. Stevens. *In Biology:* J. R. Gething.

L.D.S.—*Preliminary Science Examination. In Biology:* D. C. Bodenham, A. J. H. Orchard, D. M. Sanders, J. G. Windmill.

APPOINTMENTS.

**Royal College of Surgeons.**—Hunterian Professor: MacDonald Critchley, M.D., F.R.C.P. Fifth MacLoghlin Scholarship: Joseph Francis Smith (Bristol Grammar School).

---

**British Journal of Children's Diseases,** Vol. 16, No. 3 (1919). —The above part wanted to complete set in Library.

